# Association between the retinal age gap and systemic diseases in the Japanese population: the Nagahama study

**DOI:** 10.1007/s10384-025-01205-3

**Published:** 2025-04-30

**Authors:** Takuro Kamei, Masahiro Miyake, Keina Sado, Kazuya Morino, Yuki Mori, Yasuharu Tabara, Fumihiko Matsuda, Hiroshi Tamura, Akitaka Tsujikawa

**Affiliations:** 1 Department of Ophthalmology and Visual Sciences, Kyoto University Graduate School of Medicine, 54 Kawahara-cho Syogoin Sakyo-ku, Kyoto, 606-8507 Japan; 2 Department of Ophthalmology, Osaka City General Hospital, Osaka, Japan; 3 Center for Genomic Medicine, Graduate School of Medicine, Kyoto University, Kyoto, Japan; 4 Center for Innovative Research and Education in Data Science, Institute for Liberal Arts and Sciences, Kyoto University, Kyoto, Japan

**Keywords:** Retinal age gap, Nagahama Study, Biomarker, Systemic health, Deep learning

## Abstract

**Purpose:**

To investigate the retinal age gap, defined as the difference between deep learning-predicted retinal age and chronological age, as a potential biomarker of systemic health in the Japanese population.

**Study design:**

Prospective cohort study.

**Methods:**

Data from the Nagahama Study, a large-scale Japanese cohort study, were used. Participants were divided into fine-tuning (n=2,261) and analysis (n=6,070) cohorts based on their visit status across the two periods. The fine-tuning cohort only included individuals without a history of systemic or cardiovascular diseases. A deep learning model, originally released in the Japan Ocular Imaging Registry, was fine-tuned using a fine-tuning cohort to predict retinal age from images. This refined model was then applied to the analysis cohort to calculate retinal age gaps. We conducted cross-sectional and longitudinal analyses to examine the association of these gaps with systemic and cardiovascular diseases.

**Results:**

The retinal age-prediction model achieved a mean absolute error of 3.00–3.42 years. Cross-sectional analysis revealed significant associations between the retinal age gap and a history of diabetes (β = 1.08, p < 0.001) and hyperlipidemia (β = –0.67, p < 0.001). Longitudinal analysis showed no significant association between the baseline retinal age gap and disease onset. However, onset of hypertension (β = 0.35, p = 0.049) and hyperlipidemia (β = 0.34, p = 0.035) showed marginal associations with an increase in retinal age gap over time.

**Conclusion:**

The retinal age gap is a promising biomarker for systemic health, particularly in relation to diabetes, hypertension, and hyperlipidemia.

**Supplementary Information:**

The online version contains supplementary material available at 10.1007/s10384-025-01205-3.

## Introduction

Chronological age is a major risk factor for fatal conditions such as stroke and lifestyle-related diseases such as diabetes and hypertension [1–3]. However, significant health disparities among people of the same age have been noted, leading to increased interest in the concept of biological age as a more accurate reflection of an individual’s health status [4]. Various methods to quantify biological age have been developed, including epigenetic markers based on DNA methylation patterns and imaging techniques such as brain scans and facial analysis [5–9]. Although these approaches offer new insights into the aging process, they face challenges related to cost, ethical considerations, and invasiveness. 

The retina, being an extension of the central nervous system, reflects systemic health, and its condition can indicate both ocular and systemic diseases. Advancements in deep learning demonstrate its ability to predict various factors from retinal diseases like retinitis pigmentosa to chronological age, using retinal images [10–12]. Previous studies show that a larger retinal age gap, defined as the difference between retinal age predicted by deep learning and chronological age, can serve as a biomarker for higher mortality rates and an increased risk of diseases, such as Parkinson’s disease [11, 12]. These findings suggest that the retinal age gap may capture underlying biological aging processes that are not apparent through chronological age alone.

Initial studies on the retinal age gap were conducted using data from the UK Biobank [11, 12], and a Korean group has reported a similar concept called RetiAge [13]; our study provides additional insights into the relationship between the retinal age gap and systemic diseases in the Japanese population.

## Materials and methods

The study adhered to the tenets of the Declaration of Helsinki. The Nagahama Study and the procedures used to obtain informed consent were approved by the Ethics Committee of the Kyoto University Graduate School and Faculty of Medicine and the Nagahama Municipal Review Board of Personal Information Protection. All participants received an explanation of the purpose and methods of the study and provided informed consent. Patient data were anonymized prior to analysis.

### Study population

**Fig. 1:**
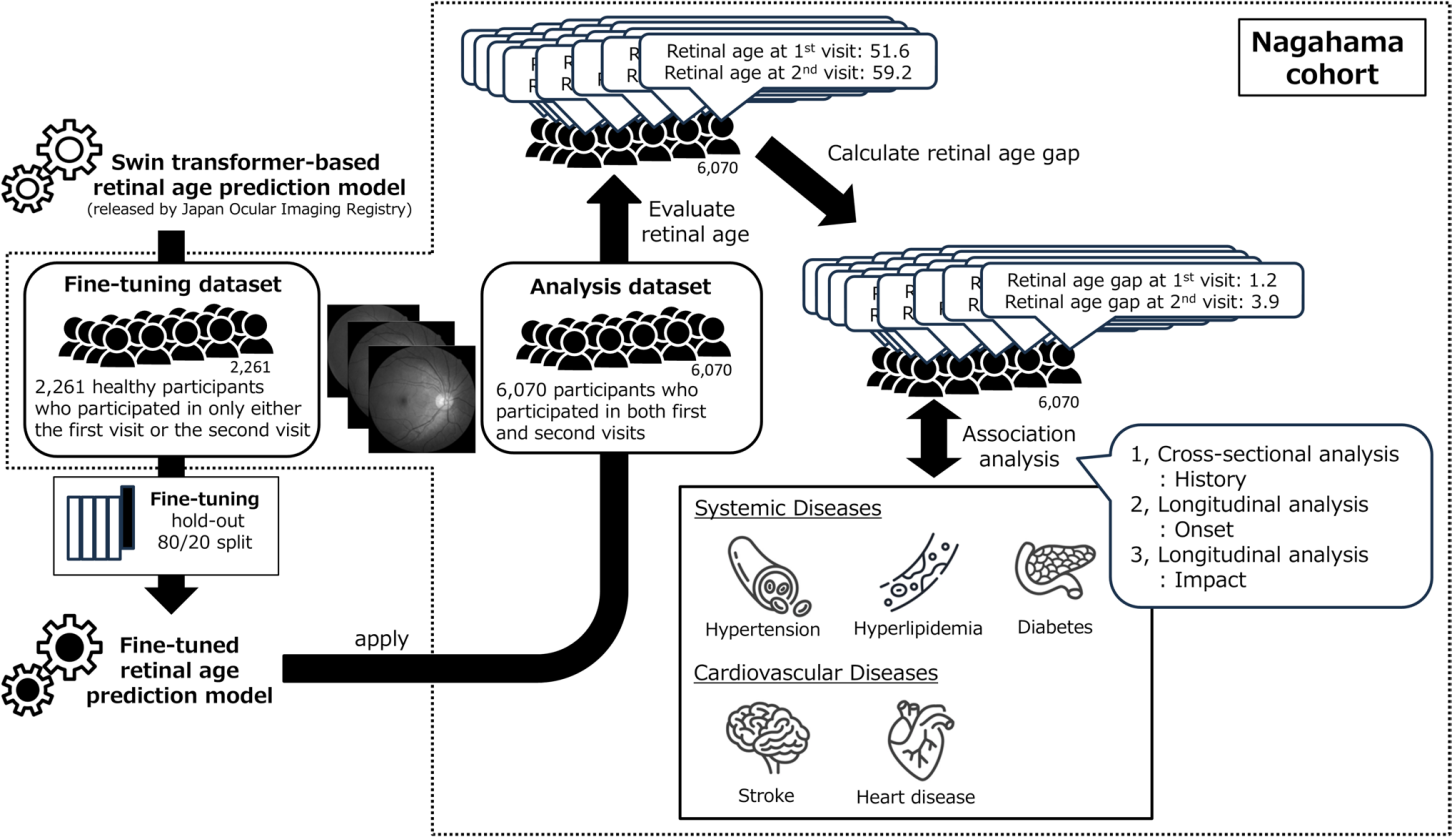
Workflow of retinal age prediction and analysis in the Nagahama Study. This figure illustrates the process of retinal age prediction and its association with systemic diseases and cardiovascular diseases, using data from the Nagahama Cohort Study. The workflow begins with a swine-transformer-based retinal age prediction model released by the Japan Ocular Imaging Registry, which serves as the foundation for this study. The Nagahama cohort is then divided into two datasets: a fine-tuning dataset consisting of 2,261 healthy participants who attended only one visit (either first or second) and an analysis dataset comprising 6,070 participants who attended both visits. The initial model undergoes fine-tuning using the fine-tuning dataset, with a hold-out 80/20 split for model validation. Subsequently, a fine-tuned retinal age prediction model is applied to the analysis dataset. The model generates predictions for each participant, with an example output showing predicted retinal ages of 51.6 years at the first visit and 59.2 years at the second visit, along with calculated retinal age gaps of 1.2 years at the first visit and 3.9 years at the second visit. The final phase of the study involves three types of analyses to investigate the relationship of the retinal age gap with systemic diseases and cardiovascular diseases. These include a cross-sectional analysis examining the history of diseases, a longitudinal analysis predicting the onset of diseases, and a longitudinal analysis assessing the impact of disease onset on changes in the retinal age gap

This study utilized data from the Nagahama Prospective Genome Cohort for Comprehensive Human Bioscience (the Nagahama Study), a large-scale population-based cohort study conducted in Nagahama, Japan. Although details of the Nagahama Study have been described elsewhere [14–18], we provide a concise overview here. In the study, detailed health information and ophthalmological measurements were collected over multiple years, making the study an ideal resource for investigating the relationship of the retinal age gap with systemic and cardiovascular diseases. Color fundus photographs were obtained from all participants (CR-DG10; Canon), and histories of systemic diseases and cardiovascular diseases were obtained using a questionnaire. All information about these systemic and cardiovascular diseases was collected through a standardized self-administered questionnaire at each visit. The presence of each disease was determined solely based on participants' self-reported responses to these questionnaires. For this study, we focused on participants who had undergone retinal imaging during the first (2008–2010) and second visit (2013–2015) of the study. An overview of the data processing and model development is shown in Figure 1. We divided the participants into two groups based on their participation in the Nagahama Study. One group consisted of individuals who participated in either the first or second visit of the Nagahama Study; this group was used for fine-tuning the publicly available deep learning model (fine-tuning dataset, n = 3,589). The other group consisted of those who participated in both visits; this group was used to evaluate the association of the retinal age gap with systemic and cardiovascular diseases (analysis dataset, n = 6,070).

### Deep learning model to evaluate retinal age

To construct a retinal age prediction model for the current study, we fine-tuned an existing deep learning retinal age prediction model. We employed the PyTorch library in Python3 using our fine-tuning dataset. The existing model, based on the Swin Transformer architecture, was created by the Japanese Ophthalmological Society, National Institute of Informatics, and the Japan Ocular Imaging Registry (JOIR) and is publicly available (http://www.joir.jp/data/en_index.html) [19–21]. Full details are provided on the website, in brief, this publicly available model was developed for age prediction using the fundus images of 12,734 healthy individuals. The model reportedly achieved a mean absolute error (MAE) of 2.39 years.

For the fine-tuning process, we only used healthy individuals, defined as those with no history of hypertension, hyperlipidemia, diabetes, stroke, or heart disease (n = 2,261). The input images were resized to 384 × 384 pixels and the model was trained for 100 epochs with a batch size of 16. Data augmentation techniques, including random vertical flipping, color jitter, random rotation, random erasing, and Gaussian blurring, were applied. The mean squared error was used as the loss function, and the Adam optimizer was employed with a learning rate of 1e-4. In addition, a cosine-annealing learning rate scheduler with a warmup was implemented to manage the learning rate during training.

The performance of the deep learning model was evaluated using the MAE. The data are shown as the mean ± standard deviation. The relationship of the retinal age gap with systemic diseases and cardiovascular diseases was assessed using regression coefficients and their 95% confidence intervals (CIs), along with p-values, to determine statistical significance. All analyses were performed using Python 3.8.0 and Pytorch 1.9.0 [19].

### Calculation of the retinal age and retinal age gap and attention map creation

The fine-tuned retinal age prediction model was applied to retinal images from the analysis group. We included patients aged <70 years on their second visit, as the original JOIR model was developed using a dataset with limited representation of older individuals, which could affect the reliability of predictions for people aged ≥70 years. Based on previous studies, the retinal age gap was calculated by subtracting the chronological age from the predicted age [11, 12, 22, 23]. Attention maps were created using gradient-weighted class activation mapping (Grad-CAM) to visualize the areas of the retina on which the artificial intelligence model focused for age prediction [24]. Following a previous study on attention visualization in transformer-based model, we specifically targeted the normalization layer of the last block in the last layer of the network to generate activation maps for our model [25]. To implement this, we used the PyTorch Grad-CAM library, which is publicly available on GitHub [26].

### Association of the retinal age gap with systemic diseases and cardiovascular diseases

Three analyses were conducted to explore the relationship of the retinal age gap with systemic diseases and cardiovascular diseases. First, we performed a cross-sectional analysis using first-visit data from the analysis dataset to determine whether individuals with a larger retinal age gap had a history of diseases. The association between the retinal age gap and the history of each systemic disease (i.e., hypertension, hyperlipidemia, and diabetes) and cardiovascular diseases (i.e., stroke and heart disease) was assessed using a univariable model and two multivariable linear regression models. The first multivariable model was adjusted for sex and age and the second was adjusted for sex, age, and smoking history.

Second, we conducted a longitudinal analysis to investigate whether the retinal age gap at baseline could predict the onset of disease over a 5-year follow-up period. The occurrence (y=1) or nonoccurrence (y=0) of each disease was used as the dependent variable. The occurrence was defined as new cases of each condition diagnosed between the first and second visits. Participants who had reported having the condition at the first visit were excluded from this analysis for that specific condition. Multivariable logistic regression analysis was used to evaluate these associations, with adjustments for sex, age, and smoking history.

Third, we analyzed the impact of disease onset on changes in the retinal age gap over 5 years. Multivariable regression analysis was conducted to assess whether the onset of systemic diseases contributed to an increase in the retinal age gap between the first and second visits after adjusting for sex, age, and smoking history. The change in the retinal age gap (i.e., retinal age gap at the second visit minus that at the first visit) was used as the dependent variable.

## Results

### Fine tuning

The fine-tuning dataset consisted of 2,261 healthy individuals, with a mean chronological age of 49.1 ± 11.8 years. There were 742 men (32.8%) and 1,519 women (67.2%). The dataset was randomly split into training and validation sets, with 1,808 and 453 images used for training and validation, respectively. The mean chronological ages of the training set and validation set were 49.3 ± 11.8 years and 48.4 ± 11.6 years, respectively. The MAE for the validation set of 453 images was 3.28 years, with a correlation coefficient of 0.922.

### Epidemiology of retinal age

**Fig. 2:**
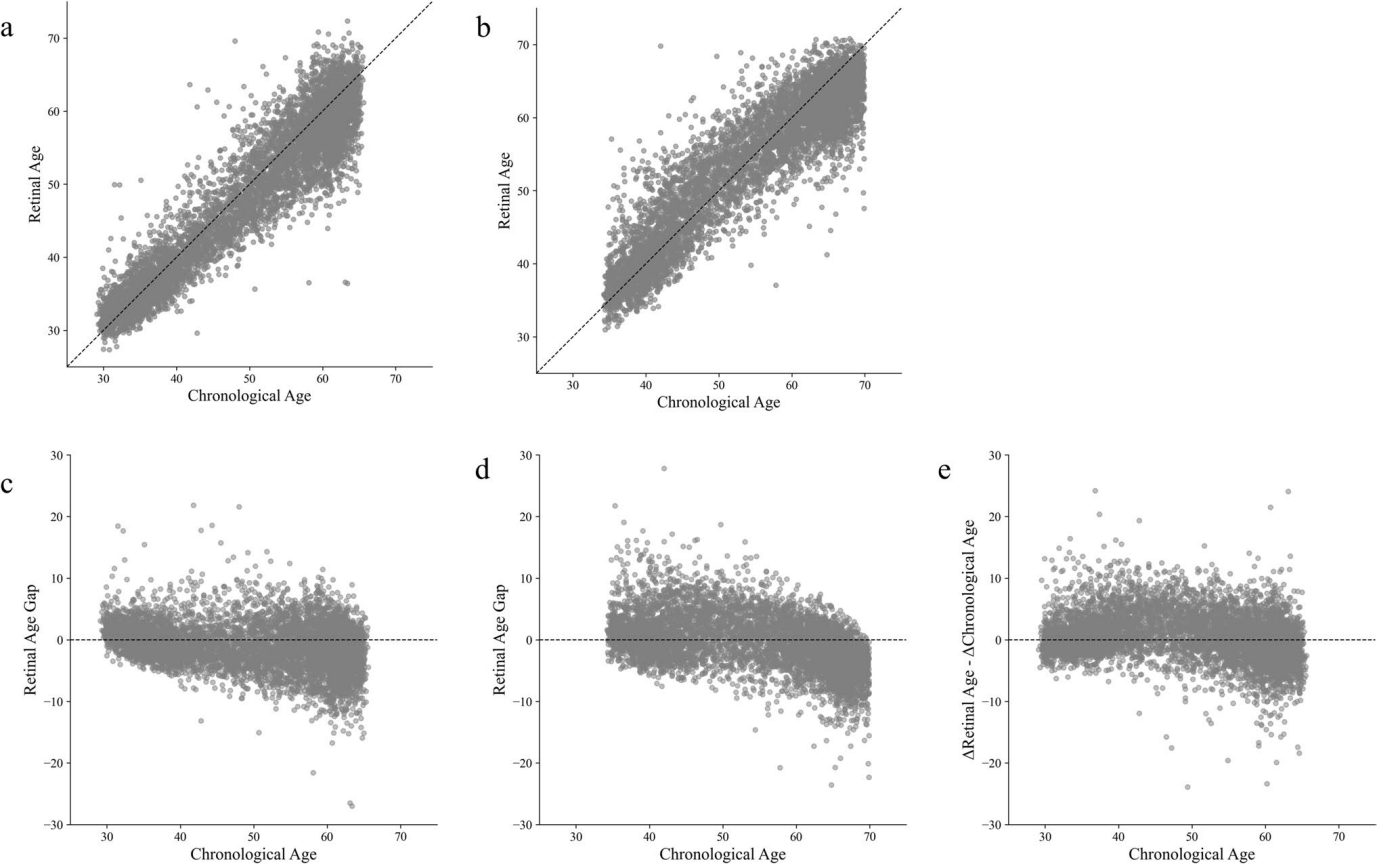
Retinal age analysis across two time periods. This figure illustrates the progression and comparison of the retinal age metrics across the two time periods in the study cohort. **a** relationship between chronological and retinal age at the first visit. **b** distribution of the retinal age at the second visit. **c** the retinal age gap (i.e., the difference between the retinal and chronological age) at the first visit. **d** retinal age gap at the second visit. **e** difference between the change in retinal age and change in chronological age from the first to the second visit (Δ_retinal age_ – Δ_chronological age_)

The analysis dataset consisted of 6,070 individuals, including 1,727 men (28.5%) and 4,343 women (71.5%). The mean chronological age was 49.1 ± 11.1 years at the first visit and 54.0 ± 11.0 years at the second visit. The mean retinal age predicted by the fine-tuned retinal age prediction model was 48.1 ± 10.6 years for the images of the first visit and 53.6 ± 9.9 years for the images of the second visit. The mean absolute difference between the retinal age and chronological age was 3.00 years for images from the first visit and 3.42 years for images from the second visit. Overall, retinal age was highly correlated with chronological age (R^2^ = 0.87 for the first visit data, and R^2^ = 0.84 for the second visit data, Figure 2a–d). Because of the 5-year interval between the 1st and 2nd visits, the chronological age increased by approximately 5 years from the first visit to the second visit. The difference between this increase in chronological age (Δ_chronological age_) and the corresponding increase in retinal age over the same period (Δ_retinal age_), namely Δ_retinal age_ – Δ_chronological age_, was 0.55 ± 3.96 years (Fig. 2e). Supplemental Figure 1 shows examples of retinal images and attention maps that compare the first and second visits of the same individuals. The deep learning model primarily focused on areas around the fovea and retinal blood vessels when making age predictions.

### Association between the retinal age gap and history of systemic diseases and cardiovascular diseases

Table 1 presents the association between the retinal age gap and systemic conditions at baseline. Hypertension, hyperlipidemia, and diabetes were found to have nominally significant associations with the retinal age gap. However, after adjusting for age and sex, the significant association between hypertension and the retinal age gap disappeared. In contrast, both hyperlipidemia and diabetes remained significantly associated with the retinal age gap even after adjusting for age, sex, and smoking history (β = –0.67, 95% CI: –0.98 to –0.36, p < 0.001, and β = 1.08, 95% CI: 0.56–1.59, p < 0.001, respectively).

**Table 1:** Association between retinal age gap and prevalence of diseases

Disease	Plain		Sex+age		Sex+age+smoking history	
	β (95% CI)	*p*Value	β (95% CI)	*p*Value	β (95% CI)	*p*Value
Hypertension	–0.821 (–1.106 to –0.536)	<0.001	–0.007 (–0.292 to 0.278)	0.963	–0.006 (–0.291 to 0.279)	0.967
Hyperlipidemia	–1.192 (–1.511 to –0.873)	<0.001	–0.669 (–0.980 to –0.358)	<0.001	–0.668 (–0.979 to –0.357)	<0.001
Diabetes	0.532 (0.004 to 1.060)	0.048	1.081 (0.568 to 1.595)	<0.001	1.077 (0.563 to 1.591)	<0.001
Stroke	0.707 (–0.763 to 2.177)	0.346	0.924 (–0.494 to 2.343)	0.202	0.919 (–0.499 to 2.338)	0.204
Heart Disease	–0.475 (–1.141 to 0.190)	0.161	0.111 (–0.533 to 0.755)	0.736	0.107 (–0.538 to 0.751)	0.746

*CI* confidence interval

### Association between the baseline retinal age gap and the onset of systemic diseases and cardiovascular diseases

Table 2 shows the association between the baseline retinal age gap and the onset of systemic diseases and cardiovascular diseases. The baseline retinal age gap was not significantly associated with the onset of any of the diseases evaluated, including hypertension (odds ratio [95% CI] = 1.00 [0.97–1.02], p = 0.657), hyperlipidemia (odds ratio [95% CI] = 0.99 [0.97–1.01], p = 0.490), diabetes (odds ratio [95% CI] = 1.02 [0.98–1.06], p = 0.341), stroke (odds ratio [95% CI] = 1.03 [0.96–1.10], p = 0.429), and heart disease (odds ratio [95% CI] = 0.97 [0.91–1.03], p = 0.261).

**Table 2:** Association between retinal age gap and occurrence of diseases

Disease	Occurrences between visits	Odds Ratio (95% CI)	*p*Value
Hypertension	527	0.995 (0.971—1.018)	0.657
Hyperlipidemia	682	0.993 (0.971—1.014)	0.490
Diabetes	150	1.020 (0.979—1.062)	0.341
Stroke	55	1.026 (0.962—1.095)	0.429
Heart Disease	54	0.965 (0.905—1.027)	0.261

*CI* confidence interval

### Impact of disease onset on the retinal age gap

Table 3 shows the association between disease onset and the changes in retinal age between the first and second visits (Δ_retinal age_). The results show that the onset of hypertension (β = 0.35, 95% CI: 0.002–0.70, p = 0.049) and hyperlipidemia (β = 0.34, 95% CI: 0.02–0.65, p = 0.035) were nominally significantly associated with an increase in the retinal age gap. However, the association was not statistically significant after Bonferroni correction. No significant associations were observed for the onset of diabetes (β = –0.16, 95% CI: –0.78 to 0.47, p = 0.625), stroke (β = –0.52, 95% CI: –1.54 to 0.51, p = 0.324), or heart disease (β = –0.66, 95% CI: –1.69 to 0.38, p = 0.215).

**Table 3:** Impact of disease onset on retinal age gap progression

Disease	Occurrences between visits	β (95% CI)	*p*Value
Hypertension	527	0.352 (0.002 to 0.702)	0.049
Hyperlipidemia	682	0.336 (0.024 to 0.648)	0.035
Diabetes	150	–0.156 (–0.784 to 0.471)	0.625
Stroke	55	–0.516 (–1.543 to 0.510)	0.324
Heart Disease	54	–0.656 (–1.693 to 0.381)	0.215

*CI* confidence interval

## Discussion

This study investigated the potential of the retinal age gap, defined as the difference between deep-learning-predicted retinal age and chronological age, as a biomarker for systemic health. Our analysis of the data from the Nagahama cohort provided several key insights. First, the deep learning model used in this study demonstrated a high level of accuracy, making it a reliable tool for predicting retinal age. Second, individuals with a history of diabetes had a significantly larger retinal age gap, suggesting that diabetes may accelerate retinal aging. Third, the onset of hypertension and hyperlipidemia significantly contributed to the increase in the retinal age gap over time, indicating that these conditions may have an immediate impact on retinal aging.

The MAE of the retinal age predictions in our study was 3.00 years for images of the first visit and 3.42 years for those of the second visit, comparable to the MAE reported in previous studies on retinal age prediction (3.26–3.55 years) [12, 27]. In addition, compared to various age prediction models using other organs or tissues, our model demonstrated a high level of accuracy. Brain age prediction models typically report MAEs of 3.56–9.54 years [8, 28], while facial image prediction models generally achieve MAEs in the range of 2.79–6.38 years [6, 7]. Laboratory data-based models utilizing biomarkers from blood tests show an MAE of 5.55 years [29]. This suggests that retinal age prediction models can achieve a precision that is comparable to or even better than models used for other organs, reinforcing the potential of retinal imaging as a valuable biomarker for systemic health assessment. Although there was an average period of 4.9 years between the first and second visits, our model maintained accuracy in tracking the same individuals over time (Fig. 2e). This suggests that retinal age prediction models are accurate at a single point in time as well as useful for longitudinal assessments, highlighting their potential for tracking changes in retinal age over extended periods.

A previous study reports that Hb_A1c_ can be predicted using fundus imaging [27]. This study focused on blood vessels, optic discs, and other non-specific features when predicting Hb_A1c_ from fundus images. In this study, the finding that the retinal age gap was significantly associated with a history of diabetes but not with the onset of diabetes itself suggests that long-term, rather than short-term diabetes, may contribute to overall retinal aging, even in the presence of diabetes treatment. Conversely, while the onset of hypertension and hyperlipidemia was significantly associated with an increase in the retinal age gap, a history of these conditions did not significantly elevate the retinal age gap. Moreover, a history of hypertension showed no significant association, and a history of hyperlipidemia was associated with a significant decrease in the retinal age gap. These findings imply that hypertension and hyperlipidemia may induce retinal aging through mechanisms such as vascular stress and inflammation; however, these effects are likely short-term and could be reversible with appropriate treatment. Indeed, treatment of patients with hypertension and hyperlipidemia, such as co-administration of amlodipine and atorvastatin, is reported to lead to improvements in arterial compliance [30]. Therefore, it is plausible that the appropriate treatment could halt the progression of retinal aging, as well as reduce retinal age by alleviating vascular stress and inflammation in the retina.

The absence of a significant association between the baseline retinal age gap and the occurrence of diseases contrasts with previous studies that identified the retinal age gap as a predictive biomarker for stroke and cardiovascular disease [22, 23]. In our study, this discrepancy may be attributed to the relatively small number of incident cases, the shorter follow-up period, and potential ethnic differences. Further replication studies are warranted to clarify whether the retinal age gap can serve as a reliable biomarker for the onset of ischemic stroke and cardiovascular disease.

Nevertheless, the retinal age gap could serve as a useful biomarker for identifying individuals at a higher risk of accelerated aging and associated systemic diseases. Regular retinal imaging and deep-learning-based retinal age prediction can be integrated into routine clinical practice to monitor retinal health and potentially identify early signs of systemic diseases. For individuals with a large retinal age gap, targeted interventions such as lifestyle modifications, stricter disease management, and closer monitoring may be warranted to mitigate the risks of accelerated aging and related complications. Additionally, our findings suggest that monitoring changes in the retinal age gap over time could provide insights into the progression of systemic diseases and treatment effectiveness. For example, an increase in the retinal age gap despite treatment may indicate the need for more aggressive or alternative therapeutic strategies.

The strengths of this study include the use of a large, well-characterized cohort with longitudinal data, and the application of a fine-tuned deep learning model for retinal age prediction. These comprehensive data allowed us to adjust for important covariates and conduct a robust statistical analysis. However, this study has some limitations. First, we did not account for the duration of each condition, which may influence the retinal age gap. Second, our medical history data relied entirely on self-reported questionnaires without verification against medical records or clinical documentation. While self-reported medical history is widely used in epidemiological studies, the possibility of recall bias or misclassification cannot be excluded. Third, owing to the limited sample size and follow-up period, we did not observe sufficient incident cases of stroke and cardiovascular diseases, which might limit the statistical power. Future studies with larger sample sizes and longer follow-up periods may elucidate the effect of the baseline retinal age gap in predicting stroke or cardiovascular events in Asian populations. Fourth, although the onset of hypertension and hyperlipidemia significantly increased the retinal age gap, this association was not statistically significant after correction for multiple tests. Therefore, they may represent false positives. Finally, a key limitation of our study is the lack of comparison with other biological age markers such as telomere length and DNA methylation age. Future studies incorporating these measurements would be valuable to validate retinal age gap as a biological aging marker and understand its underlying mechanisms. Despite these limitations, we believe that our findings offer novel insights into the association between the retinal age gap and systemic diseases.

In conclusion, our study highlights the potential of the retinal age gap as a biomarker of systemic health, particularly in relation to diabetes, hypertension, and hyperlipidemia. These findings underscore the importance of integrating retinal age prediction into clinical practice to monitor retinal health and identify individuals at risk of accelerated aging and related systemic diseases. Regular monitoring of the retinal age gap could help guide clinical interventions and evaluate treatment effectiveness in patients with systemic diseases.

## Supplementary Information

Below is the link to the electronic supplementary material.

Supplementary file1 (PDF 52 KB)
