# Supplementary material for: Association between the retinal age gap and systemic diseases in the Japanese population: the Nagahama study
Source: Jpn J Ophthalmol. 2025 Apr 30;69(4):616–23. doi: 10.1007/s10384-025-01205-3 (PMC12339582; doi:10.1007/s10384-025-01205-3)
Supplement: Supplementary file 1 — Supplementary file1 (PDF 52 KB) [file 10384_2025_1205_MOESM1_ESM.pdf]

Supplementary Material

Article Title: Association Between the Retinal Age Gap and Systemic Diseases in the Japanese Population: the Nagahama Study

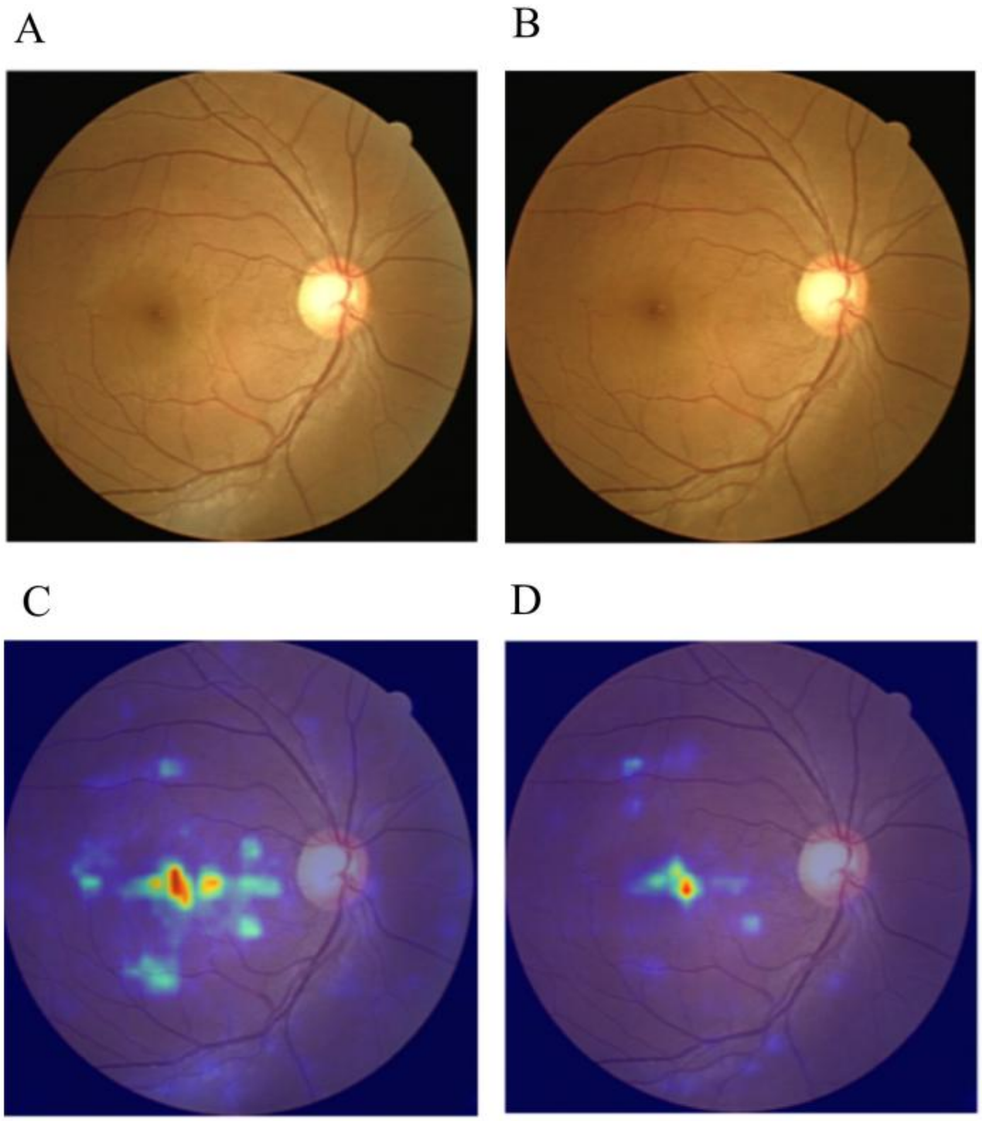

Supplemental Fig. 1 Example of changes in the predicted retinal age from the first visit to the second visit. This figure shows an example of changes in predicted retinal age for the same individual between the first and second visits. (a) represents the original retinal image from the

first visit, while (b) shows the original retinal image from the second visit. (c) displays the attention map for the first visit, indicating the areas of the retina on which the deep learning model focused on for age prediction. (d) shows the attention map for the second visit. The chronological and predicted ages for the first visit are 38.6 and 38.3 years, respectively, and for the second visit, they are 43.3 and 42.1 years, respectively. Although no significant differences were observed by the naked eye between the first and second visits, the predicted retinal age changed. The attention maps show the focal points around the fovea and blood vessels.
